# The impact of teacher’s presence on learning basic surgical tasks with virtual reality headset among medical students

**DOI:** 10.1080/10872981.2022.2050345

**Published:** 2022-03-09

**Authors:** Sofianna Ojala, Joonas Sirola, Timo Nykopp, Heikki Kröger, Henrik Nuutinen

**Affiliations:** a University of Eastern Finland and Kuopio University Hospital, Kuopio, Finland; b Kuopio Musculoskeletal Research Unit (KMRU), Kuopio, Finland

**Keywords:** VR, virtual reality, HMD, head-mounted display, surgical education, teaching methods

## Abstract

**Background:**

The aim of this study was to investigate whether the presence of a teacher affects learning related outcomes in teaching basic surgical tasks with a Virtual Reality (VR) headset.

**Methods:**

26 fourth-year medical studentsparticipated in a voluntary exercise. Students practiced basic surgical procedure exercises using the VR4HEALTHCARE application in VR with OCULUS Rift S glasses. 12 students performed the exercises under the guidance of a teacher and 14 without the teacher present. After the exercise, the groups filled out a feedback form. Statistical analysis was performed using IBM SPSS Statistics 25.0 software using the Mann-Whitney U test and multivariate analysis of variance.

**Results:**

The most important data collected related to whether the student learned something new and whether VR adds value to medical education. Ratings were based on a scale of 0–10 (0 = worst, 10 = best). When the teacher was present, on average, the students felt that they were learning something new and gave an average rating of 7.8 ± 1.8, and when the teacher was not present 5.3 ± 2.6 (p = 0.003). VR added value to teaching with a rating of 7.8 ± 1.7 when the teacher was present and 5.5 ± 3.0 when not present (p = 0.045). This study also analyzed specific use of VR for abscess incision, suturing and insertion of a suprapubic catheter.

**Discussion:**

When a teacher was present VR added value to teaching and the usefulness and usability of VR was experienced more positively. The student should also have adequate knowledge of the subject to be taught before VR training.

**Conclusions:**

VR adds value to teaching, but VR exercises may not completely replace high-quality traditional teaching methods. Consequently, it is important to determine the differences between VR and traditional teaching methods and how to combine these methods in the future.

## Introduction

Virtual Reality (VR) allows the user to learn and operate in computer-generated environments in real time to gain hands-on experience that can be used later in clinical work [1,2]. VR was first used in healthcare in the early 1990s to visualize complex medical structures during surgeries and preoperatively in planning surgeries [2]. The most studied VR application is screen-based display, also known as simulation. In this study, however, we focus on HMD (head-mounted display), which is a less-studied form of VR in medical teaching. There are many publications on training with a VR simulator, but only a few on training with HMD. VR simulation has been shown to enhance clinical and surgical training [1]. However, there are also some drawbacks associated with simulators, such as lack of realistic haptics (feeling in VR), that may be challenging with respect to immersion [3].

Based on previous studies, VR exercises can improve professional self-confidence [4]. Gamification in learning can be utilized with the current generation of learners as various console games have been played since childhood. It can also increase motivation to learn, but in the other hand it can be expensive and difficult to incorporate into the curriculum[5]. Peden conducted a prospective randomized study with 14 students randomized to three groups: conventional teaching, HMD-assisted teaching and HMD self-learning. The study showed that in learning basic surgical tasks, in this case suturing, students preferred HMD-assisted teaching to conventional teaching alone. However, there were no differences in surgical skill level [6]. Also, the presence of a teacher was assessed as more useful in previous studies, than only self-learning in virtual reality [7,8]. As an example, teacher can advise what to do during the task to make the student feel more confident. The teacher can also advise and help with the uncertainty associated with the new technology, making its use more comfortable.

The aim of this study was to examine how the presence of a teacher affects learning when the teaching happens with VR glasses. The aim of this study was not to study the improvement in surgical skills as such. The hypothesis of this study is that the presence of a teacher in VR exercises improves self-estimated usefulness of VR teaching of basic surgical tasks.

## Methods

First, a research question was set i.e., whether the presence of a teacher affects self-estimated learning. A research form was then developed (which is available as an appendix in Supplement 1). The questions were based on the authors experience in medical teaching, but was not based on any validated questionnaire.

In this study, 80 fourth-year medical students at the University of Eastern Finland were sent an email informing them of the opportunity to participate in this voluntary VR exercise. The students gave informed consent for study participation. The questionnaire was formulated based on the pedagogic competence of the teacher (HN) and included questions aimed at developing teaching in VR environments (Supplement 1). 26 fourth-year medical students wanted to participate in this voluntary exercise. The exercises were done between March and December 2019.

**Figure 1.**
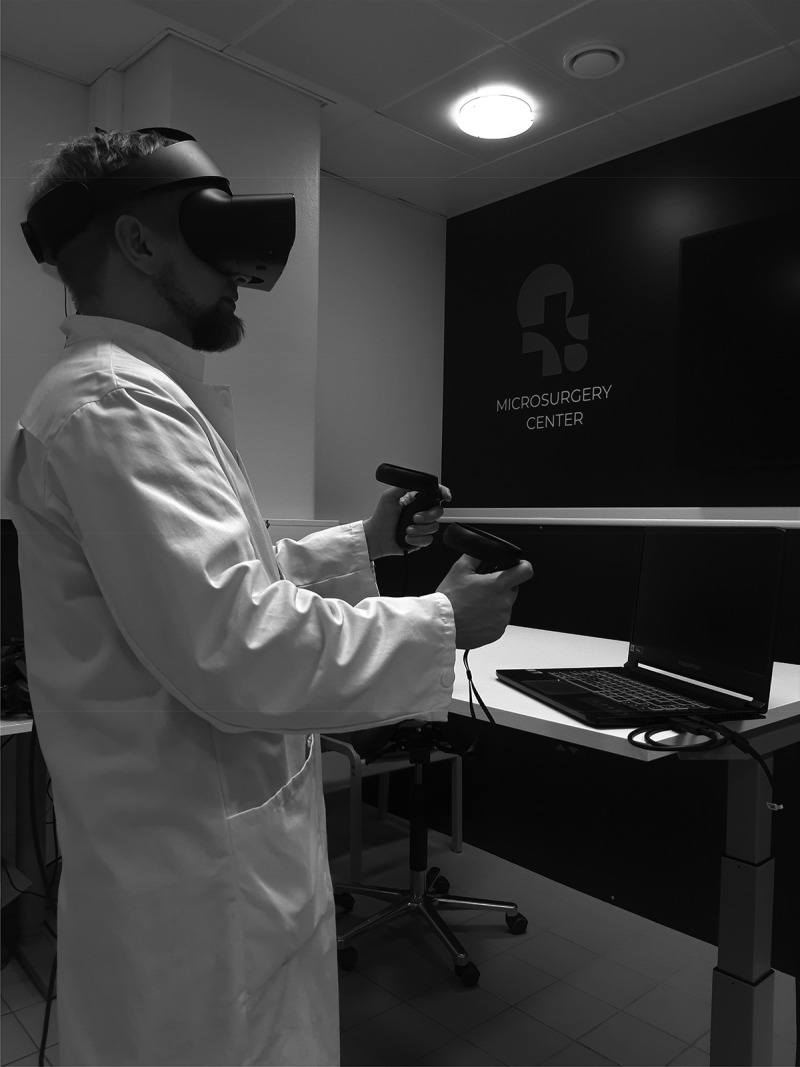
Set-up of the exercise.

Students were not given preliminary assignments and the topics of the exercises were not known to the students in advance. Students practiced basic surgical tasks procedures and exercises using the VR4HEALTHCARE application in VR with OCULUS Rift S glasses. The exercise set-up is shown in Figure 1. The exercises were suturing, abscess incision and suprapubic catheter insertion. Prior to the exercise, an initial introduction to the use of the device was given similarly to both groups. Twelve (12) students completed the exercises under the guidance of the teacher and fourteen (14) students completed the exercises without the guidance of the teacher. Students were allowed to choose available times from the electronic appointment system. The teacher was present in the afternoon sessions. However, students were blinded to the presence of the teacher at the time of booking. After the exercise, students filled out a feedback form (Supplement 1). The collected data included whether the student learned something new, whether VR adds value to medical education, or whether VR was useful when learning suturing, inserting a suprapubic catheter or incising an abscess with or without a teacher’s presence. These tasks were rated on a scale of 0 to 10 (0 = worst, 10 = best). Statistical analysis was performed using IBM SPSS Statistics 25.0 software. We used Fisher’s exact test to compare nominal data and the Mann-Whitney U-test for nonparametric data. Results were considered statistically significant at p-values < 0.05. Multivariate analysis of variance (MANOVA) was used to study the effect of covariates on the main outcome. The present study was not a randomized controlled trial. Naturally, the presence of the teacher cannot be blinded.

According to the 488/1999 Finnish Medical Act (amendment 295/2004, 794/2010) research related to the development of teaching quality does not require permission from the research ethics committee.

## Results

24 students were right-handed and two students were left-handed (Table 1). Genders were quite equally represented with 12 men and 14 women (Table 1). The mean age was 29.2 years in the group with the teacher present and 24.9 years in the group without the teacher present, and age was statistically significantly different (p = 0.025) between these two groups (Table 1). 11 students had used VR before (Table 1). The Modes of the present study were: age 24, usefulness of the exercise 9, usability of exercise 7, learned something new 8, abscess incision 8, insertion of suprapubic catheter 8, suturing 8 and VR added value to teaching 8. The analysis was performed with the absolute means.

**Table 1.** Characteristics and main results of the effect of teacher’s presence on selected outcomes (n = 26)

	Teacher present (mean ± SD)	Teacher not present (mean ± SD)	P value^a^	P value^b^
Basic information: Answers (n)	12	14		
Sex			0.713	
*Male*	5	7		
*Female*	7	7		
Age (years)	29.2 ± 6.7	24.9 ± 2.6	0.025	
Handedness (n)			1.000	
*Right*	11	13		
*Left*	1	1		
Have used VR before (n)			0.233	
*Yes*	7	4		
*No*	5	10		
Learning outcomes related to use of VR:				
Usefulness of the exercise*	8.3 ± 1.4	5.3 ± 2.4	0.001	p < 0.001
Usability of the exercise*	7.3 ± 1.7	6.0 ± 1.7	0.060	p = 0.015
Learned something new*	7.8 ± 1.8	5.3 ± 2.6	0.003	p = 0.001
VR added value to teaching*	7.8 ± 1.7	5.5 ± 3.0	0.045	p = 0.016
Technical usability of VR:				
The exercise fit physically, mentally and educationally (n)			0.225	
*Yes*	12	11		
*No*	0	3		
Usability for teaching abscess incision*	8.0 ± 1.2	6.0 ± 3.1	0.079	
Usability for teaching suturing*	7.4 ± 1.4	2.6 ± 3.0	0.006	
Usability for teaching suprapubic catheter insertion*	8.5 ± 1.1	5.4 ± 3.2	0.007	

*On a scale of 0–10 (0 = worst, 10 = best)

a) Nonparametric data, nominal data

b) Significance in multivariable analysis of variance for learning related outcomes.

When the teacher was present, the students felt, on average, that they were learning something new with a rating of 7.8 ± 1.8, and when the teacher was not present with a rating of 5.3 ± 2.6; there was a statistically significant difference (p = 0.003) (Table 1). Students felt that VR added value to teaching with a rating of 7.8 ± 1.7 with the teacher present and 5.5 ± 3.0 when the teacher was not present (p = 0.045) (Table 1). The usefulness of the exercise was perceived as good when teacher was present with a rating of 8.3 ± 1.4, but when teacher was not present at the exercise the usefulness was perceived as worse with a rating of 5.3 ± 2.4 (p = 0.001) (Table 1). Usability of the exercise was perceived as the same whether the teacher was present or not with a rating of 6.0–7.3 ± 1.7 (p = 0.060) (Table 1). We also analyzed the effect of presence of the teacher on learning related outcomes (usefulness and usability of the exercise, learned something new, VR added value to teaching) using multivariable analysis of variance (Table 1). The model was adjusted for the covariates listed in Table 1, Basic information. All of the outcome variables were statistically significant. The coefficient of determination (R [2]) was 0.56 for usefulness of the exercise, 0.29 for usability of the exercise, 0.45 for learned something new and 0.36 for VR added value to teaching. Previous use of VR was the only significant covariate for usefulness of the exercise. None of the other covariates showed statistical significance.

Three students felt that VR is not a good fit for them physically, mentally or educationally. The biggest reason was nausea due to the VR-glasses (Table 1). In this study, specific uses of VR were also tested. These included abscess incision, suturing and insertion of a suprapubic catheter. Usability of VR for these specific cases was perceived as good when the teacher was present with a rating of 7.4–8.5 ± 1.1–1.4 (Table 1). When teacher was not present, the usability of teaching incision of an abscess was still considered quite good with a rating of 6.0 ± 3.1 (p = 0.079), but the usability for teaching suturing was perceived as quite poor with a rating of 2.6 ± 3.0 (p = 0.007) (Table 1). Usability for teaching suprapubic catheter insertion without a teacher present was perceived as neither bad nor good with a rating of 5.4 ± 3.2 (p = 0.007) (Table 1).

## Discussion

This study showed that, when a teacher was present, use of a VR headset added value to teaching and the usefulness and usability of VR was experienced more positively. This result supports previous observations that learning related outcomes are much better when a teacher is present [7,8].

HMD has three components, i.e., visual, hearing and immersion, and thus cannot be compared to traditional screen-based techniques or lecture teaching. This study adds to current knowledge on situations in which immersive HMD techniques could be used and utilized in teaching. The previous study of Peden used Google glasses, which are not fully immersive, meaning that the surrounding environment of the user is seen [6]. Our study also brings new knowledge to areas that the research by Peden did not examine, especially regarding a specific use of VR in surgical procedures[6]. All medical students should have competency in these procedures in the Finnish medical curriculum, and it has been found to be challenging to teach these with traditional teaching methods. In the present study, suprapubic catheter insertion with VR glasses was perceived as the most beneficial experience especially in the group with a teacher present during the exercise. The abscess incision exercise in VR was rated almost as good as suprapubic catheter insertion. Suturing received the weakest rating with regard to these specific exercises, but still quite good usability was experienced with the teacher present versus without the teacher present during the exercise. Some written critical feedback was given about suturing because the feeling in VR (haptics) does not compare well with the real feeling of suturing.

An earlier publication by Dankbaar found that inexperienced students did not benefit from serious or overly complex procedures [9]. In this present study, we can see the same effect when the teacher was not present in performing surgical procedures. This study also shows that it may be possible to teach things that cannot be taught effectively using traditional teaching methods.

The participants were however not aware whether the teacher was present or not during the exercise. This was a single-blind non-randomized controlled trial. 80 4th-year medical students were invited, and all students willing to participate were included. Naturally, the willingness to participate in this VR exercise may be a source of selection bias, considering the history of use of such technology. Those who did not want to participate may not have been willing to come to VR training. The opposite may also be the case; those who had never seen VR glasses before may have wanted to try them. In addition, no power calculation was performed due to lack of validated outcome variables.

The authors believe that VR teaching should be introduced in the following manner. The learning theory behind this is cognitive experiential [10,11]. Students must first have a good level of theoretical knowledge. Theory and basic information are learned from books and lectures, and this knowledge is deepened in small group lessons and in seminars. The next step after the theory part is to learn how things are done in practice. This part increases the level of students’ experience. These also have a significant effect on increasing professional self-confidence and, later, the comfort level of work. Uncomfortable working leads to burnout over time. Once the theory and knowledge of how things work in practice is acquired, one can move on to practicing in VR. VR presents an opportunity to practice procedures in the real environment before treating real patients, and it could make complex procedures easier to learn without exposing real patients to extra risks [3]. In addition, there is no pressure to succeed when practicing in VR. VR exercises also improve professional self-confidence [4,12]. As mentioned above, these active teaching methods have been shown to be more effective than passive ones [13]. Further, gaining expertise cannot be achieved passively [^14–16^]. The authors consider active methods in this context to include observing more experienced colleagues, traditional simulations, VR, and watching videos. After the basics of procedures are learned in VR, the next step is practicing on a real patient. However, in the beginning this needs to be done under supervision. When comparing VR teaching to situations without VR teaching, real patients are at higher risk due to lack of experience and practice without former VR experience.

This study has a few limitations to be considered. One of the limitations of the present study was small number of participants. Although the invitation e-mail was sent to 80 students, the number of students willing to participate remained unfortunately small. By comparison, the corresponding Peden study had only 14 participants and the meta-analysis of Guedes had 695 participants across 20 studies (16 to 84 students per study) [6,17]. Another possible limitation was that the groups were not fully comparable in their characteristics; previous use of VR and teacher presence were randomized and not pre-arranged. Further, the students who participated in this research could be more interested in surgery than students who did not participate, and therefore the study group may not be representative of the general population of medical students. The last limitation to be considered is that the rating scale (values 0 to 10) in the questionnaire was not a validated scale (such as Likert), which may have increased the tendency for selecting 0 or 10 values. However, this scale has been widely used before [18,19]. In addition, wider scale may provide more differences as outcome variable in studies with small sample size.

## Conclusions

VR adds value to teaching, but VR exercises may not completely replace high-quality traditional teaching methods. Consequently, it is important to determine the differences between VR and traditional teaching methods and how to combine these methods to best effect in the future.

## Supplementary Material

Supplemental Material

## Declarations

The datasets used and/or analyzed during the current study are available from the corresponding author on reasonable request.
