# Supplementary material for: The impact of teacher’s presence on learning basic surgical tasks with virtual reality headset among medical students
Source: Med Educ Online. 2022 Mar 9;27(1):2050345. doi: 10.1080/10872981.2022.2050345 (PMC8920371; doi:10.1080/10872981.2022.2050345)
Supplement: Supplemental Material [file ZMEO_A_2050345_SM4084.docx]

**Supplement 1**

**Feedback form: VR (virtual reality) -exercise**

**Participant: Male Female Other/don’t want to say**

**Age: _____ Handedness: R / L**

**Have you used VR before: Yes No**

**General questions of the exercises:**

Usefulness 0-10 (0= useless, 10= very useful): _____

Usability 0-10 (0= difficult to use, 10= very workable): _____

Did you learn something new 0-10 (0= nothing, 10= very much): _____

**Exercise specific questions:**

1. Abscess incision:

Usability for teaching 0-10 (0= worst, 10= best): _____

1. Suturing:

Usability for teaching 0-10 (0= worst, 10= best): _____

1. Suprapubic catheter incision:

Usability for teaching 0-10 (0= worst 10= best): _____

**VR added value to teaching 0-10 (0= none, 10= very much):** ______

**The exercise fit physically, mentally and educationally for me**

**Yes No**

**Free word and development ideas: _______________________________________________________________________________________________________________________________________________________________________________________________________________**
